# A Case Report of Peritoneal Mesothelioma as an Acute Abdomen Mimic: A Rare Presentation and Diagnostic Challenges

**DOI:** 10.7759/cureus.74598

**Published:** 2024-11-27

**Authors:** Lana Fadl, Mohammed Fadl, Ola Fadl, Siddalingana Gouda Thaplar G. Gouda, Hibah Mirza

**Affiliations:** 1 Internal Medicine, Walsall Healthcare NHS Trust, Walsall, GBR; 2 General Practice, Mersey and West Lancashire Teaching Hospitals NHS Trust, Birmingham, GBR; 3 Internal Medicine, The Royal Wolverhampton NHS Trust, Wolverhampton, GBR; 4 Medicine, Walsall Healthcare NHS Trust, Walsall, GBR; 5 Microbiology, Walsall Healthcare NHS Trust, Walsall, GBR

**Keywords:** acute abdomen, asbestos, diagnosis, malignant, peritoneal mesothelioma, thrombosis

## Abstract

Malignant peritoneal mesothelioma (MPM) is a rare and aggressive cancer often linked to asbestos exposure. This case report presents a 60-year-old man with a history of asbestos exposure who developed MPM, initially presenting with acute abdominal pain, an uncommon mimic of the acute abdomen. Diagnosing MPM is challenging due to its vague symptoms, often leading to delayed diagnosis. Additionally, the patient developed internal jugular vein thrombosis, a rare complication associated with malignancies. This case highlights the rare presentation of peritoneal mesothelioma as an acute abdomen mimic, the diagnostic complexities associated with MPM, and the rare type of thromboembolic event in this case.

## Introduction

Peritoneal mesothelioma is an uncommon and highly aggressive cancer that develops in the peritoneum, the membrane lining the abdominal cavity, and covering organs such as the liver and intestines. Like other forms of mesothelioma, this cancer is often associated with previous exposure to asbestos, a hazardous material that can pose health risks if inhaled or ingested. Diagnosing peritoneal mesothelioma is challenging due to its tendency to spread extensively within the abdominal lining before detection [1].

In the United Kingdom, approximately 2,700 people are diagnosed with mesothelioma each year, with men at a higher risk than women. Most cases occur in individuals aged 75 and older. Although there is currently no cure for mesothelioma, treatment options are available to help alleviate symptoms and improve quality of life [2]. Peritoneal mesothelioma likely accounts for no more than 7%-10% of all mesothelioma cases, although the exact numbers are unknown. It is much less common than pleural mesotheliomas [3].

Peritoneal mesothelioma often presents with nonspecific symptoms, such as abdominal pain, bloating, and fluid accumulation, which cause discomfort due to pressure on surrounding organs. Other signs may include changes in bowel habits, weight loss, fever, fatigue, nausea, and night sweats, with rare cases progressing to bowel obstruction. Since symptoms typically appear 20 to 40 years after asbestos exposure and are often vague or intermittent, diagnosis is frequently delayed [4].

## Case presentation

In early April 2024, a 60-year-old man who had previously worked in building trades and had a history of asbestos exposure presented to the hospital with sudden, severe central abdominal pain and nausea. His past medical history included a duodenal perforation, myocardial infarction, and insulin-dependent diabetes, and he had quit smoking several years earlier with no significant family history. His occupational history included working in a building with asbestos more than 30 years ago and, more notably, in a foundry where asbestos ropes were used. These ropes were sometimes draped around workers' necks or even held in their mouths for convenience.

**Figure 1:**
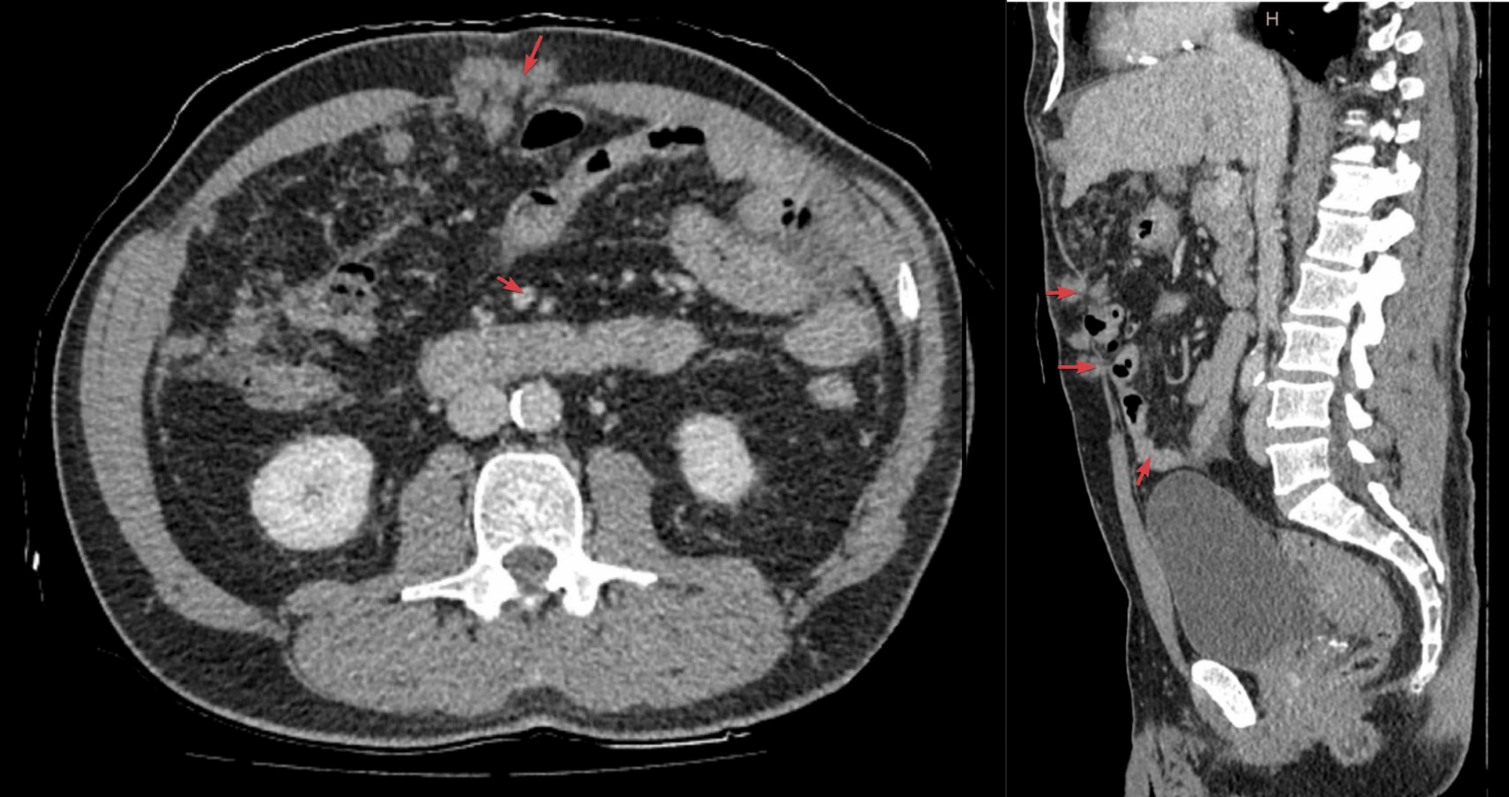
CT abdomen and pelvis with contrast (May 4, 2024) demonstrating extensive omental and peritoneal deposits. The arrows indicate areas of omental and peritoneal deposits.

**Figure 2:**
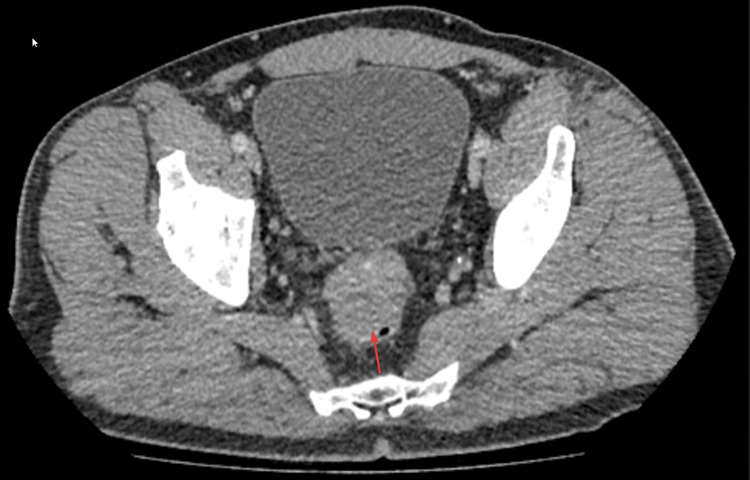
CT abdomen and pelvis with contrast done on May 4, 2024, showing a mass in relation to the upper aspect of the rectum at the rectosigmoid junction. The arrow indicates a 38.8 mm mass near the rectosigmoid junction distinct from the prostate and seminal vesicles and involving the bowel wall.

The initial examination revealed abdominal guarding and signs of peritonism. Blood tests were unremarkable except for an elevated C-reactive protein (CRP) of 130 mg/dl (reference range: 0.0-5.0 mg/dl). Suspecting an acute abdominal condition, the surgical team took over his care and arranged an urgent contrast-enhanced CT scan of the abdomen and pelvis (Figures 1, 2) to rule out perforation.

CT findings showed extensive omental and peritoneal deposits throughout the abdomen and pelvis, predominantly in the right peritoneal fossa. Serosal deposits were also noted, including those at and just above the umbilicus, within a hernia. A 38.8 mm mass was seen near the rectosigmoid junction, distinct from the prostate and seminal vesicles and involving the bowel wall. Free fluid was observed in the upper abdomen near the liver and spleen, but no other significant bowel-related mass, bowel obstruction, free gas, collections, or destructive bone lesions were identified. These findings strongly suggested metastatic disease within the abdomen and pelvis, although the primary source was unclear, with suspected bowel or peritoneal origin. A follow-up contrast-enhanced CT scan of the chest showed no evidence of lung involvement.

Further history revealed no previous episodes of abdominal pain, weight loss, nausea, or night sweats. The patient mentioned mild abdominal bloating, which he had attributed to poor dietary habits. After excluding a surgical cause, the patient was referred to the colorectal multidisciplinary team (MDT), who recommended a flexible sigmoidoscopy. Performed on April 24, 2024, the sigmoidoscopy revealed external compression at the rectosigmoid junction. No biopsies were taken as the mucosal surface appeared normal, and the report suggested a submucosal lesion, leading to the recommendation for an MRI of the pelvis.

**Figure 3:**
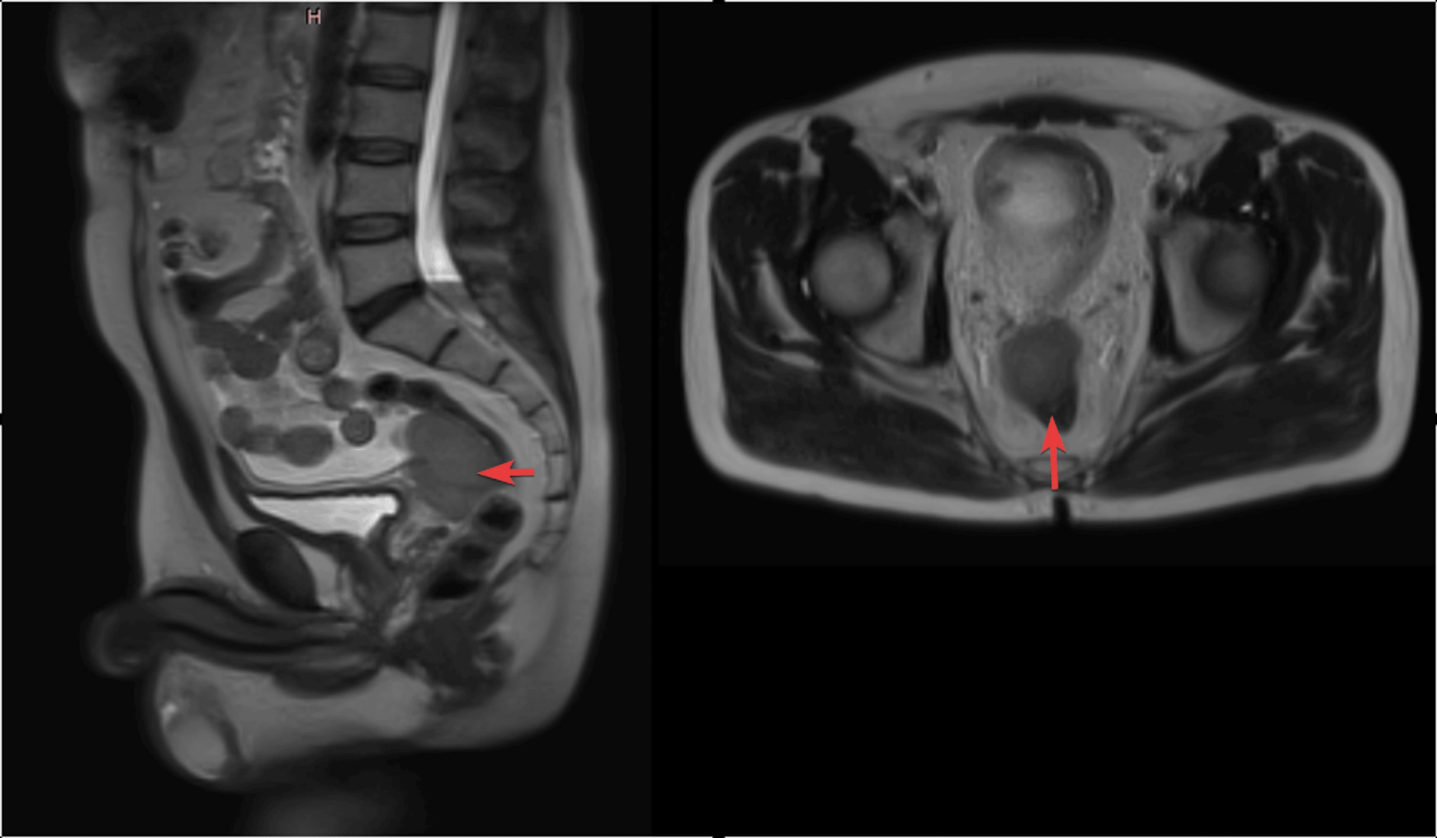
The MRI of the pelvis with contrast (August 5, 2024) revealed a soft tissue lesion in the rectovesical space, potentially indicative of a peritoneal or serosal deposit. The arrows indicate a soft tissue lesion in the rectovesical space; the lesion was external to the rectum and rectosigmoid junction.

On May 8, 2024, an MRI of the pelvis with contrast (Figure 3) showed a soft tissue lesion in the rectovesical space, likely representing a peritoneal or serosal deposit. The lesion was external to the rectum and rectosigmoid junction, with no primary tumor identified.

The MDT recommended an ultrasound-guided biopsy of the umbilical lesion, and histopathological examination confirmed epithelioid mesothelioma, leading to a final diagnosis of malignant peritoneal mesothelioma (MPM).

**Figure 4:**
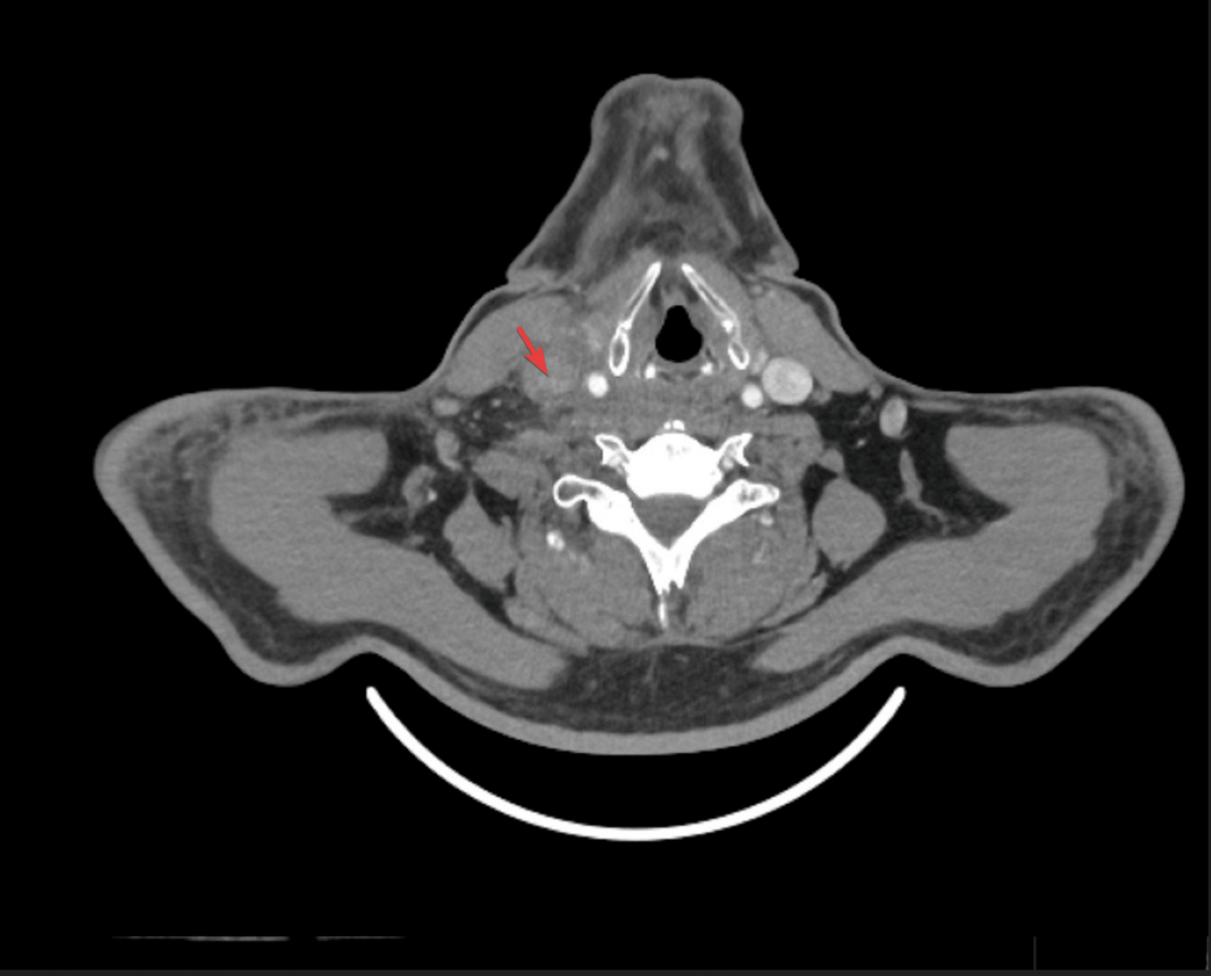
CT neck, chest, abdomen, and pelvis (June 18, 2024) (horizontal view). The arrow indicates a lack of contrast opacification in the right internal jugular vein, with surrounding fat stranding, suspicious of thrombosis.

The patient was referred to the peritoneal MDT, who decided to initiate immunotherapy with ipilimumab and nivolumab. He continued to experience right iliac fossa pain, so a low-residue diet was advised to minimize the risk of bowel obstruction. On June 18, 2024, the patient presented with right-sided neck pain, which was exacerbated by movement. An examination identified a palpable, firm mass on the right side of the neck. Laboratory investigations indicated a D-dimer level of 1147.0 ng/ml (normal range: 0-500 ng/ml). A contrast-enhanced CT scan of the neck, chest, abdomen, and pelvis (Figure 4) demonstrated a lack of contrast opacification in the right internal jugular vein, with surrounding fat stranding, suggesting the presence of a thrombus. After consulting with the vascular team, anticoagulation therapy was initiated.

**Figure 5:**
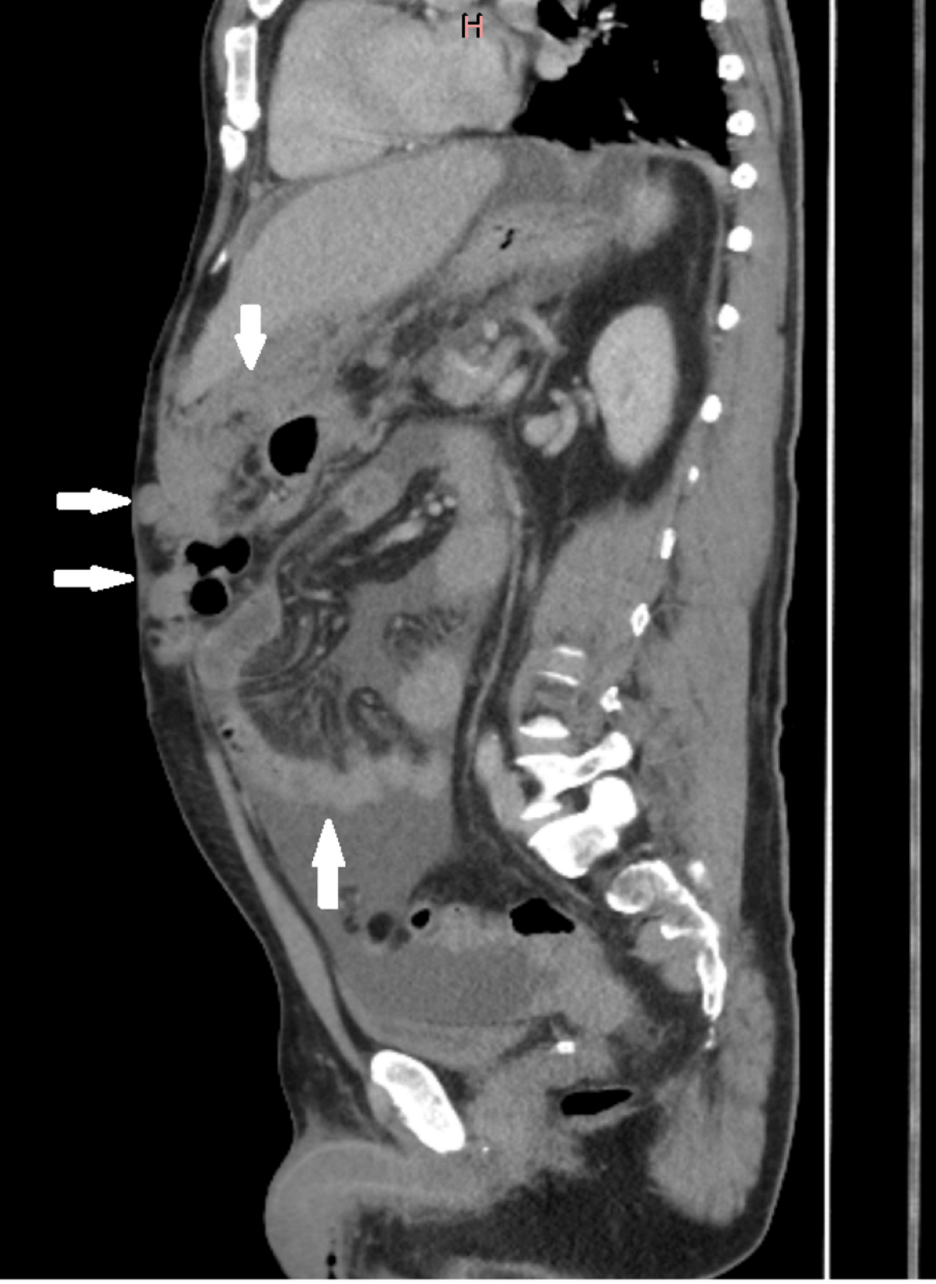
CT abdomen and pelvis (November 6, 2024): progression of peritoneal and omental deposits. The arrows indicate areas of omental and peritoneal deposits.

The patient remains on immunotherapy, with his most recent hospital visit on November 6, 2024, for abdominal distention and pain. A CT scan of the abdomen and pelvis with contrast (Figure 5) revealed progression of peritoneal and omental deposits, along with ascites. He was admitted, and 4.2 L of fluid was drained for symptomatic relief. The patient is currently under the care of the palliative team and awaiting further oncology appointments to determine the next steps in his treatment.

## Discussion

MPM is a rare primary tumor that affects the peritoneal lining. Although it shares similar epidemiological and pathological features with the more common pleural mesothelioma, it occurs less frequently. Patients often present with symptoms such as weight loss, anorexia, abdominal pain, distension, and fever of unknown origin. Additionally, symptoms related to hypercoagulability, typically linked to paraneoplastic syndromes, may also occur [5]. The occurrence of acute abdomen as the first symptom is exceptionally rare [6]. While abdominal pain is common in peritoneal mesothelioma, it is typically nonspecific and seldom presents so acutely [7], making this presentation, which resembled a surgical abdomen and initially required surgical input, unusual.

Given the rarity of MPM and its nonspecific symptoms, diagnosing the condition is challenging. This difficulty often leads to delays in diagnosis, and by the time MPM is detected, most patients already have advanced disease, significantly worsening their prognosis [8].

A definitive diagnosis of MPM in this case required extensive investigation, including imaging, flexible sigmoidoscopy, and ultimately an ultrasound-guided biopsy, after initially the rectosigmoid lesion was misidentified as a possible colorectal cancer, leading to a delay in diagnosis. This highlights the diagnostic challenges of peritoneal mesothelioma, as the disease is often widespread at the time of presentation.

Internal jugular vein (IJV) thrombosis is an extremely rare vascular condition, most commonly caused by intravenous drug abuse, prolonged central venous catheter use, or severe head and neck infections or trauma. While thromboembolic events in abdominal malignancies are common, malignancy-related IJV thrombosis is rare and not widely documented [9]. In this case, the IJV thrombosis can be attributed to the hypercoagulable state associated with peritoneal mesothelioma, which may be linked to paraneoplastic syndromes [10].

## Conclusions

This case underscores the rare presentation of MPM as an acute abdomen mimic, highlighting the significant diagnostic challenges associated with this condition. The patient's acute abdominal pain led to extensive investigation, ultimately revealing the diagnosis. Additionally, the development of IJV thrombosis, a rare complication seen in malignancies and attributed to the hypercoagulable state in MPM, adds another layer of complexity. Timely recognition and a multidisciplinary approach are crucial for improving outcomes in such rare and difficult-to-diagnose cases.
